# Right Lung Agenesis with Tracheal Stenosis due to Complete Tracheal Rings and Postpneumonectomy Like Syndrome Treated with Tissue Expander Placement

**DOI:** 10.1155/2016/4397641

**Published:** 2016-11-01

**Authors:** Yashwant Agrawal, Sandeep Patri, Jagadeesh K. Kalavakunta

**Affiliations:** ^1^Department of Internal Medicine/Pediatrics and Internal Medicine, Western Michigan University Homer Stryker School of Medicine, Kalamazoo, MI 49048, USA; ^2^Department of Cardiology, Borgess Medical Center/Michigan State University, Kalamazoo, MI 49048, USA

## Abstract

Congenital lung agenesis is an extremely rare condition with an estimated prevalence of 34 in 1,000,000 live births. It is often associated with other congenital malformations of the skeletal, cardiovascular, urogenital, and gastrointestinal systems. We discuss the case of a 5-month-old who presented with increasing stridor over 1 month. Imaging revealed right lung agenesis, complete dextromalposition of heart, and compression of distal trachea. An intrathoracic saline tissue expander was placed which marked improved distal tracheal stenosis. In patients who are symptomatic it becomes imperative to perform surgeries to increase survival as was the case in this patient.

## 1. Introduction

Congenital lung agenesis is a rare condition with a mortality rate as high as 50% by age of 5. Initial diagnosis may be delayed as the clinical picture and imaging may mimic other common diagnosis like pneumonia with parapneumonic effusion, and so forth. Almost 50% of these patients can have associated congenital malformations most notably of the skeletal, cardiovascular, urogenital, and gastrointestinal systems. These associated anomalies along with mechanical effects on the tracheobronchial tree and esophagus from mediastinal shift can result in major morbidity to this group of patients. Tracheal stenosis can be caused simultaneously by extrinsic compression from an overstretched aorta due to mediastinal displacement and intrinsic narrowing due to complete tracheal rings as seen in this patient. Symptomatic patients will need prompt surgical management when indicated.

## 2. Case Report

**Figure 1:**
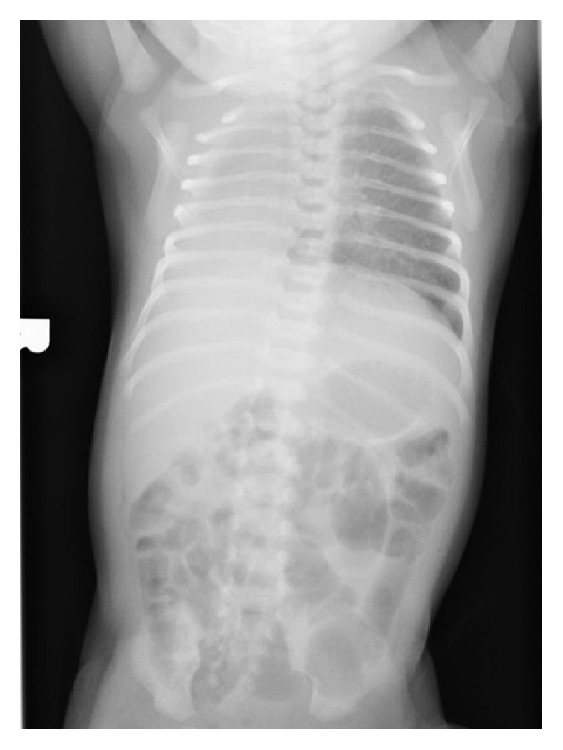
Chest X-ray revealing right lung agenesis and dextrocardia.

**Figure 2:**
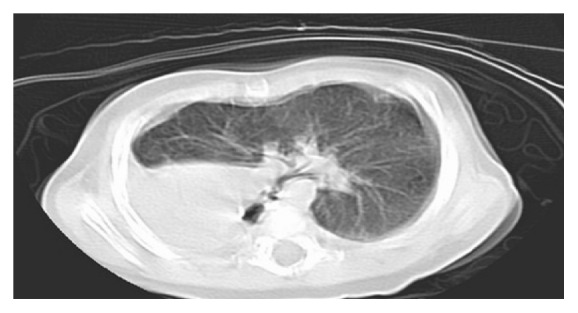
CT chest revealed right lung agenesis, dextrocardia, compression of the distal trachea, and proximal left bronchus beneath the aorta.

**Figure 3:**
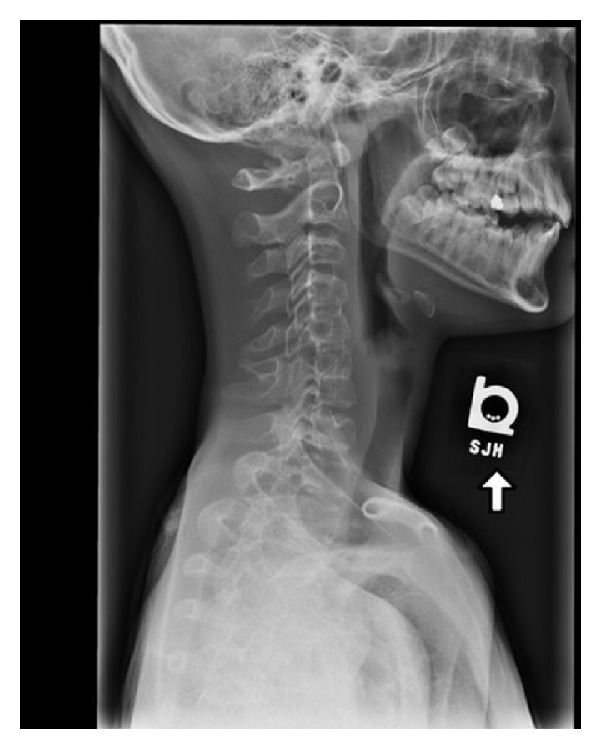
X-ray neck revealing Klippel-Feil anomaly.

We describe the case of a 5-month-old male infant who was brought to our hospital after failing multiple outpatient treatment for supposed respiratory tract infection. Since birth, he suffered recurrent episodes of respiratory distress with noisy breathing. Physical exam at our hospital revealed biphasic stridor, wheezing in the left lung fields, and transmitted upper airway sounds on the right side with dullness to percussion. Apical impulse was appreciated 2 cm inferior and medial to the right nipple and heart sounds were heard over the right anterior chest. Labs were in the normal range. Chest X-ray was interpreted as possible right-sided pneumonia with a large parapneumonic effusion (Figure 1). Computed tomography of the chest revealed right lung agenesis with resultant complete dextroposition of the heart, compression of the distal trachea, and proximal left bronchus beneath the aorta (Figure 2). Transthoracic echocardiogram was normal except for dextroposition of the heart. He had rigid and flexible bronchoscopy performed which revealed distal tracheal stenosis just above the carina. Decision was made to place an intrathoracic saline tissue expander to reposition the heart anteromedially. A saline tissue expander was placed into the extra pleural space and was inflated to 80 cc via right sixth lateral thoracotomy approach. Intraop bronchoscopy showed marked improvement in the distal tracheal stenosis and also three complete tracheal rings distally. Patient was followed up periodically with bronchoscopies which showed progressive improvement in the distal tracheal stenosis. The expander was filled periodically for the first three years. He also has associated skeletal anomalies like scoliosis and Klippel-Feil anomaly (Figure 3). He is being followed up at the pulmonary and cardiology offices annually for the past 4 years and has been doing well with achievement of normal milestones throughout.

## 3. Discussion

Pulmonary agenesis is an extremely rare malformation with an estimated frequency between 1/10,000 and 1/15,000 autopsies with an earlier report showing a prevalence of 0.0034% of all hospital admissions [1]. This can occur during the embryonic phase of the development of pulmonary tree between weeks 3 and 7 [2] but an etiology has not been established yet. Three types of lung agenesis have been described, with our patient being of type 2, described as complete absence of pulmonary parenchyma with a rudimentary bronchus [3].

Only few cases have been documented to have a normal life span, as many of the patients have associated malformations in other organ systems which increase their mortality [4–6]. Gabarre et al. found a 50% mortality rate by the age of 5 years in the patient cohort [7]. 50% of patients with right lung agenesis also have anomaly affecting at least one other organ system. These include the gastrointestinal, renal, skeletal, and most importantly the cardiovascular system [6]. It may also be part of various genetic syndromes. Anomalies in our patient included complete tracheal rings, Klippel-Feil anomaly, bilateral cervical ribs, and scoliosis.

There were two major causes for respiratory distress in this case, one being the mediastinal shift caused by the unilateral lung agenesis and other being the presence of tracheal rings causing tracheal stenosis. The major cause was the detrimental effect on the tracheobronchial tree from massive mediastinal shift to the diseased side. It is similar to a postpneumonectomy syndrome where the aorta can get stretched over and compress the trachea, bronchus, or the esophagus [8]. This can also result in herniation of the normal lung to the diseased side with compensatory hyperinflation and air trapping in that lung. The tracheal narrowing can cause recurrent respiratory infections, retention of secretions, and stridor and, if severe, lead to respiratory compromise which warrants prompt surgical management. Our patient successfully underwent tissue expander placement which significantly relieved the tracheal stenosis. There are only four other cases where this procedure has been successfully performed [5, 9]. The fact that our patient is currently having reasonable exercise tolerance with a single lung about 4 years after the procedure shows that it may be a good option for these patients. However further studies with more longitudinal follow-up have to be done to establish it. Aortopexy can be performed to relieve the extrinsic tracheal compression but will not address the other issues caused by the mediastinal shift [10, 11]. Aortopexy was not needed in our patient as the tissue expander placement successfully relieved the respiratory distress in the patient. Diaphragm translocation has also been reported to successfully correct the mediastinal shift [12].

Our case also had distal tracheal rings which further contributed to the tracheal stenosis. Complete tracheal rings by themselves are rare and usually present in the first year of life [13]. However, in a retrospective study by Backer et al. 15% out of their 71 patients with tracheal stenosis had the combination of complete congenital tracheal rings and unilateral lung agenesis or severe hypoplasia [14]. This suggests that there might be strong enough association between the two conditions to suggest that we should actively look for any form of pulmonary hypoplasia in patients with complete tracheal rings. Depending on the symptom severity, patients can be managed conservatively or may undergo surgery, most commonly a tracheoplasty [14, 15]. Our patient did not require surgery because his airway calibre progressively increased with growth and his symptoms resolved.

## 4. Conclusion

Unilateral lung agenesis should be high in the differential in any patients presenting with repeated chest infections and unilateral lung opacification. There should be a low threshold to obtain a chest X-ray and/or other imaging studies in patients presenting with recurrent pneumonias and/or stridor. Clinicians should carefully look for the myriad of possible associated defects especially cardiac and other tracheobronchial tree anomalies which can contribute to morbidity and mortality. In patients who are symptomatic it becomes imperative to perform surgeries to relieve extrinsic airway compression, prevent further hyperinflation of the healthy lung, and thereby increase survival as was the case in our patient.
